# Approach selection for resection of petroclival meningioma

**DOI:** 10.3171/2022.1.FOCVID21252

**Published:** 2022-04-01

**Authors:** Christina Jackson, Kent S. Tadokoro, Eric W. Wang, Georgios A. Zenonos, Carl H. Snyderman, Paul A. Gardner

**Affiliations:** Departments of 1 Neurological Surgery and; 2 Otolaryngology, University of Pittsburgh Medical Center, Pittsburgh, Pennsylvania

**Keywords:** endoscopic endonasal approach, anterior petrosectomy, petroclival meningioma, contralateral transmaxillary, transpterygoid

## Abstract

Petroclival meningiomas are surgically challenging tumors because of their deep location and involvement of critical neurovascular structures. A variety of approaches have been described, and selection of approach should be tailored to the location of the tumor relative to neurovascular structures and surgical experience. The authors present two patients with petroclival meningiomas with varying relationships to cranial nerves and skull base anatomy who underwent endoscopic endonasal and open petrosectomy approaches, to demonstrate the complementarity of the endonasal transpetrous and open transpetrosal corridors. Proficiency in both open and endonasal approaches is critical to appropriate approach selection and maximal safe resection.

The video can be found here: https://stream.cadmore.media/r10.3171/2022.1.FOCVID21252

## Transcript

This short series is designed to show approach selection for resection of skull base petroclival meningiomas. This approach selection should be tailored based on the location of the lesion relative to neurovascular structures. As you can see, petroclival and cavernous meningiomas can have varied localization, anywhere from medial to lateral, with different relationships.

### 0:42 Considerations of Approach Selection.

An endoscopic endonasal approach provides medial access, while transpetrosal approaches provide lateral access, making them very complementary.

### 0:53 Endoscopic Endonasal Approach (EEA) for Petroclival Meningioma.

Looking at this in slightly greater detail to understand these differences, an endoscopic endonasal approach has clear advantages, again as mentioned, for that medial access, but is limited lateral to the cavernous sinus. Access to the middle cranial fossa is also limited, and cranial nerve VI tends to be on the anterior aspect of the tumors, which can make this very challenging.

### 1:13 Adjuncts to EEA Approach.

This also requires mobilization of the carotid artery. This can sometimes be limited or minimized by applying a contralateral transmaxillary approach through the maxillary sinus to reach behind the carotid artery.

### 1:24 Anterior Petrosectomy Approach.

In contrast, an open anterior petrosectomy for petroclival meningioma gives great lateral access, all the way out to the middle fossa. But it has limited access inferior to the IAC and not as sufficient access to the full clivus; furthermore, the fourth nerve blocks our access.

### 1:43 Posterior Petrosectomy and Retrosigmoid Approaches.

A retrosigmoid approach can provide lateral access to lesions inferior to the IAC. The addition of a posterior petrosectomy can provide additional access to the middle clivus. These options provide limited access to the middle cranial fossa and anteromedial brainstem and can carry risks to hearing, facial, and venous injury.

### 2:02 Illustrative Case 1.

Here is a case of a 61-year-old woman who presents with significant imbalance and dysphagia. Her exam is normal other than her significant gait difficulties.

### 2:10 Sellar and Cavernous Sinus Exposure.

Here is the wide initial exposure, all the way from the planum down to the nasopharynx.

### 2:15 Nasopharyngeal Flap.

We will start by exposing down to the foramen magnum by doing a rhinopharyngeal flap. A needle-tip Bovie is used to dissect the rhinopharyngeal flap. This is dissected inferiorly, down to foramen magnum.

### 2:29 Paraclival Internal Carotid Artery (ICA) Exposure.

Then the paraclival carotid artery on the side of the tumor is widely skeletonized and exposed. This allows us to mobilize it and also work behind it for greater lateral access. Similarly on the left side, we do drill quite close to the paraclival carotid artery, even all the way into the jugular tubercle on that side, in order to provide a wide clival resection and access.

### 2:50 Clivectomy.

Here we can see even the periosteal layer of the dura is beginning to be removed. Here we are drilling wider on the right side and into the petrous apex and the body of the petrous bone. We have exposed the paraclival carotid arteries for pituitary transposition.

### 3:06 Petrosal Process.

Here is labeled the petrosal process of the sphenoid bone, which is a great landmark for cranial nerve VI. Here we can see the sixth cranial nerve being stimulated in an interdural fold as it enters Dorello’s canal.

### 3:19 Pituitary Transposition.

Pituitary transposition is then performed for the supratentorial portion of this tumor. The cavernous sinus is opened, and careful dissection of the pituitary gland protected by its dural sheath is performed. Here we are lifting it up and transecting the dorsum sellae to separate the two posterior clinoids, and carefully dissecting out first the right posterior clinoid. You can see the spike of bone that can often occur behind it, and eventually we also remove the left posterior clinoid for wider access.

### 3:49 Tumor Debulking.

Dura is opened in the midline, more eccentric to the contralateral side to try to avoid injury to the sixth nerve. Tumor is internally debulked. This is taken all the way up to the mid clivus. You can use two suctions for this exposure.

### 4:03

The basilar artery is then defined, and we can examine the brainstem interface very early on. You can see here it is practically indistinguishable in some areas between tumor capsule and brainstem. This is often the case with petroclival meningiomas. Two suctions alone, as well as a mini microdebriding device, can be used very carefully here to again try to avoid injury to the sixth nerve.

### 4:27 Supratentorial Tumor.

Supratentorial resection is performed by a pituitary hemitransposition all the way up to the diaphragma. Two suctions are used working on either side both above and below, and next to the pituitary gland to dissect out the basilar apex, PCA, and SCA, even up to the posterior communicating artery. Here we see that wide dissection. You can see the sixth nerve here, which has a bundle of tumor around it to try to preserve its function.

### 4:55 Indocyanine Green Angiography (ICG).

Indocyanine green angiography is performed at the end of the resection to visualize the various vascular structures and the third cranial nerve coming out of the brainstem, all of which have been protected.

### 5:08 Reconstruction.

A multilayer closure with fascia lata, fat graft, and nasoseptal flap is performed. We perform ICG also to confirm the viability of the nasoseptal flap. Here we can see postoperative imaging showing a great resection and decompression of the brainstem from this medial approach. The patient had resolution of her preoperative symptoms, now walking independently with resolution of her hydrocephalus. Given the patient’s age and improvement in symptoms, we elected for continued observation of the residual tumor with plans for possible radiosurgery if there is future growth.

### 5:42 Illustrative Case 2.

Counter to that case, here is a 32-year-old man who previously underwent a similar endoscopic endonasal approach for resection of petroclival meningioma after presenting with double vision and myelopathy, who now presents with progression and fifth and sixth nerve symptoms.

### 5:59 Middle Meningeal Artery.

Here we can see sectioning first the middle meningeal artery.

### 6:02 Ligation of Meningo-Orbital Band.

Then the meningo-orbital band to de-vascularize the region.

### 6:09 Interdural Dissection.

Then a standard interdural dissection is performed along the edge of the cavernous sinus. This is taken down toward Meckel’s cave and then finally all the way back to the middle fossa.

### 6:19 Greater Superficial Petrosal Nerve (GSPN).

Here GSPN is carefully dissected free, taken right up to V3. Once this has been done, we have completely exposed Meckel’s cave, which you can see here is bulging with tumor. We can drill out the anterior petrous bone dissecting all the way to the petrous apex.

### 6:36 Petrous ICA.

We then carefully expose the petrous carotid artery. This allows us to provide the most radical resection of the petrous apex once it has been visualized.

### 6:50 Anterior Petrosectomy.

We then procced with the petrosectomy, drilling just shy of the IAC and drilling all the way out almost to the carotid artery, taking care to preserve the cochlea.

### 7:00 Anterior Clinoidectomy.

Next, switching gears entirely. We switch over to the anterior clinoidectomy, which is performed in the usual fashion. Here you can see the last bits of the clinoid being removed.

### 7:10 Identification of V3.

V3 is then stimulated. We widely decompress V3 in its bony canal in order to be able to mobilize it during resection.

### 7:20 Decompressing Cranial Nerve (CN) V.

Opening Meckel’s cave, we see tumor simply filling Meckel’s cave, which explains the patient’s fifth nerve symptoms. Then dura is opened with both frontal and temporal flaps transected down to the roof of the cavernous sinus in the fashion described by Ali Krisht.

### 7:36 CN III.

Further dissection is performed. We identify the third nerve, which can be stimulated directly. The tumor between the third nerve and brainstem is then resected.

### 7:46 CN IV.

Next, the fourth nerve is identified at the tentorium as it crosses into the cavernous sinus. Once you identify the fourth nerve, all the tumor between the third and fourth nerve until they fully enter the cavernous sinus can be resected.

### 8:02 Meckel’s Cave.

Finally, the fifth nerve as it exits the pons at the porus trigeminus can also be identified. We can open all the way out to Meckel’s cave to fully expose the trigeminal nerve and gasserian ganglion and try to dissect tumor with as much preservation of the fifth nerve and gasserian ganglion as possible, which you can see being done here.

### 8:21 Posterior Fossa.

Finally, we expose the posterior fossa by transecting the tentorium. Resection of the tumor is taken back into the posterior fossa, where we see a glimpse of the brainstem. We are working down here medially and anteriorly to the IAC, identifying the basilar artery working just above the sixth nerve. Here we can see the final result with small residual left, of course, in the cavernous sinus itself. Here we can see the postoperative imaging showing as radical of a resection as possible without damage to the cavernous sinus nerves.

### 9:04 Conclusions.

These cases very nicely show the approach selection, which is based, of course, on patient symptoms and relief of those symptoms. Lateral approach is chosen for access to tumors lateral to cranial nerves in the lateral cavernous sinus and middle cranial fossa, whereas endonasal is preferred for medial tumors.^1–5^

## Disclosures

The authors report no conflict of interest concerning the materials or methods used in this study or the findings specified in this publication.

## Author Contributions

Primary surgeon: Gardner. Assistant surgeon: Jackson, Tadokoro, Snyderman. Editing and drafting the video and abstract: Gardner, Jackson, Tadokoro, Zenonos. Critically revising the work: Gardner, Jackson, Wang, Zenonos, Snyderman. Reviewed submitted version of the work: Gardner, Jackson, Wang, Zenonos, Snyderman. Approved the final version of the work on behalf of all authors: Gardner.
